# Nitrogen-Doped Titanium Dioxide Mixed with Calcium Peroxide and Methylcellulose for Dental Bleaching under Visible Light Activation

**DOI:** 10.3390/ijms22073759

**Published:** 2021-04-04

**Authors:** Minal Thacker, Yi-Ning Chen, Chun-Pin Lin, Feng-Huei Lin

**Affiliations:** 1 Graduate Institute of Biomedical Engineering, National Taiwan University, Taipei 10051, Taiwan; d05548019@ntu.edu.tw (M.T.); d2825065d@gmail.com (Y.-N.C.); 2 Graduate Institute of Clinical Dentistry, School of Dentistry, National Taiwan University, Taipei 10617, Taiwan; chunpinlin@gmail.com; 3 National Taiwan University Hospital, College of Medicine, National Taiwan University, Taipei 10617, Taiwan; 4 Institute of Biomedical Engineering and Nanomedicine, National Health Research Institutes, Miaoli County 35053, Taiwan

**Keywords:** bleaching, calcium peroxide, titanium dioxide, nitrogen doping, visible light

## Abstract

The available tooth whitening products in the market contain high concentrations of hydrogen peroxide (H_2_O_2_) as an active ingredient. Therefore, in order to curb the high H_2_O_2_ concentration and instability of liquid H_2_O_2_, this study evaluated the efficacy and cytotoxicity of the bleaching gel composed of 10% calcium peroxide (CaO_2_) and visible-light-activating nitrogen-doped titanium dioxide (N-TiO_2_) with methyl cellulose as a thickener. Extracted bovine teeth were discolored using coffee and black tea stain solution and were divided into two groups (*n* = 6). Bleaching was performed thrice on each tooth specimen in both the groups, with one minute of visible light irradiation during each bleaching time. The CIELAB L*a*b* values were measured pre- and post-bleaching. The N-TiO_2_ calcinated at 350 °C demonstrated a shift towards the visible light region by narrowing the band gap energy from 3.23 eV to 2.85 eV. The brightness (ΔL) and color difference (ΔE) increased as bleaching progressed each time in both the groups. ANOVA results showed that the number of bleaching significantly affected ΔE (*p* < 0.05). The formulated bleaching gel exhibits good biocompatibility and non-toxicity upon exposure to 3T3 cells. Our findings showed that CaO_2_-based bleaching gel at neutral pH could be a stable, safe, and effective substitute for tooth whitening products currently available in the market.

## 1. Introduction

The desire to flaunt a beautiful smile has turned into an aesthetic necessity today. Hence, tooth whitening has become rampant in esthetic dentistry [1]. The demand for that near-perfect smile has triggered the production of several whitening products and the success of each such product is dependent on the type of tooth discoloration [2]. Tooth discoloration can be classified into two main categories: external staining, which is mainly due to poor oral hygiene, smoking, chlorhexidine in mouthwashes, pigmented food, dental caries, or beverages; whereas internal staining is attributed to factors like age, antibiotics, or excessive fluoride content [3,4].

Hydrogen peroxide is the most common active ingredient in dental bleaching products, due to its ability to release free radicals [5]. These free radicals trigger the oxidation and splitting of pigmented organic molecules into smaller molecules. In turn, these smaller molecules reflect more light, making the tooth appear brighter and whiter, achieving a successful bleaching [6,7,8]. For vital tooth bleaching, both in-office and home bleaching techniques are widely used [9]. Office bleaching products usually contain a high concentration of H_2_O_2_ (35–40%) to generate high levels of free radicals for a short treatment time in one appointment, while home bleaching products contain 6–10% H_2_O_2_ and take more than one month to achieve desired results [10,11]. The higher H_2_O_2_ concentration or longer exposure time in contact with bleaching gel is effective in whitening. However, side effects might be generated. The most common complications include tooth sensitivity and gingival irritation [9]. With the increasing concentration of H_2_O_2_, adverse side effects, such as dental hypersensitivity, soft tissue irritation, and cytotoxicity, of dental bleach may occur. Moreover, 0.1 to 6.0% hydrogen peroxide or equivalent for hydrogen peroxide releasing from dental bleaching products is reported safe as per the European Scientific Committee on Consumer Products (SCCP) [12]. However, dental bleaching gel consisting of varying concentrations of H_2_O_2_ showed toxicity in different cell lines, such as fibroblasts and dental pulp cells (DPCs), in previous reports [13,14,15,16]. Therefore, a safer yet efficient bleaching product is long overdue.

Calcium peroxide (CaO_2_) can be a potential substitute of liquid H_2_O_2_ in dental bleaching products because of its characteristics, such as a more effective source of H_2_O_2_, and relatively stable nature than liquid H_2_O_2_. CaO_2_ is considered to be the safest form of solid peroxy compounds. Upon dissolution in water, CaO_2_ is capable of reacting in the medium for a longer time and releases H_2_O_2_ in a controlled manner. The maximum H_2_O_2_ released per gram of CaO_2_ is 0.47 g, which is considered safe in dentistry [17,18]. According to previous studies, CaO_2_ is capable of releasing H_2_O_2_ and O_2_ independently. Moreover, the releasing rates can be controlled by various factors, such as pH and temperature; that is, an increase in pH leads to a decrease in the release of H_2_O_2_ and increases the O_2_ yield, while the increased temperature improves the release of O_2_ [19].

In the past, there have been studies showing the use of high-energy ultraviolet (UV)-light-assisted catalysts to reduce the concentration of hydrogen peroxide in dental bleaching products [20]. However, UV-light imposed greater risks on the eyes and skin of the patients as well as that of the dentists [21]. To address the challenges imposed by the harmful UV light, a visible light-activating photocatalyst titanium dioxide was recently introduced. The feasibility of TiO_2_ can be attributed to its nontoxicity, low price, and photostability [22]. Titanium dioxide (TiO_2_), in its crude form, is a well-known photocatalytic material activated by UV light but can be modified to achieve photo response at visible light. Doping TiO_2_ with nitrogen turns out to be the most effective way to reduce the band gap, and transition its photo response from UV light to visible light [23,24,25].

Given this background, the focus of this study was to develop a safe and effective dental bleaching product for clinical use. This product comprises calcium peroxide and visible light-activating nitrogen-doped TiO_2_ (N-TiO_2_) as a photocatalyst to increase the rate of bleaching. The cytotoxicity and efficacy of the fabricated dental bleach were examined in vitro by co-culturing with 3T3 cells, while the effectiveness of the prepared dental bleach was further evaluated using stained bovine teeth model in vitro.

## 2. Results

### 2.1. X-ray Diffraction (XRD) Analysis

**Figure 1:**
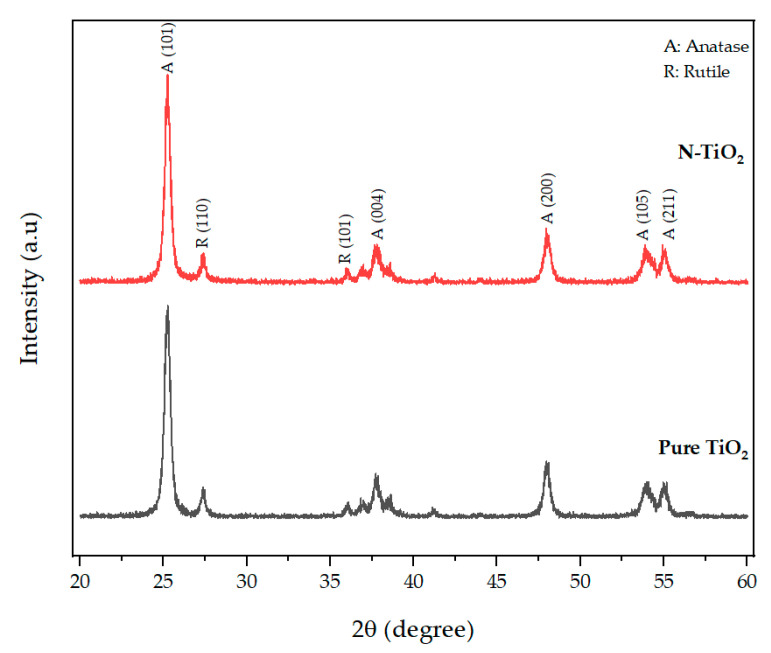
XRD patterns of TiO_2_ and nitrogen-doped TiO_2_.

XRD analysis was used to characterize the crystal phase structure of the prepared photocatalytic TiO_2_. The XRD pattern of N-TiO_2_, along with the pattern of pure TiO_2_ used as a reference, is presented in Figure 1. The crystal phase composition of pure TiO_2_ and N-TiO_2_ contained a mixture of major anatase phase and minor rutile phase. The XRD profile of N-TiO_2_ calcined at 350 °C exhibited anatase peaks at 25.25°, 37.68°, 47.94°, 53.87°, and 55.01° and were in good accordance with the (101), (004), (200), (105), and (211) peak position of anatase TiO_2_ (JCPDS 86-1157) and rutile peaks at 27.4° and 36.03°, which were in conformity with the (110) and (101) peak position of rutile TiO_2_ (JCPDS 21-1276).

### 2.2. X-ray Photoelectron Spectroscopy (XPS) Analysis

**Figure 2:**
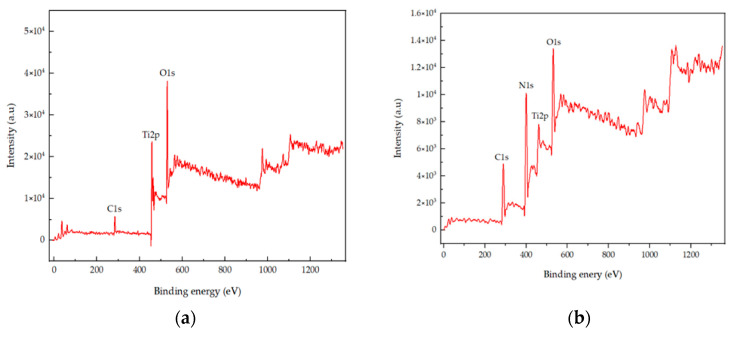
(**a**) XPS survey spectra of TiO_2_ and (**b**) XPS survey spectra of N-TiO_2_.

XPS was performed in order to characterize the chemical state and surface composition of the prepared photocatalytic TiO_2_. The spectrum of pure TiO_2_ and N-TiO_2_ is presented in Figure 2a,b, respectively. TiO_2_ predominantly contained Ti, O, and C elements, while TiO_2_ upon heat treatment indicated the presence of Ti, O, N, and C elements. Amongst these elements in N-TiO_2_, the C1s peak was located at 288.6 eV, and it represented the contaminated residual precursor, which was not completely removed during heat treatment. Moreover, accidental carbon during the process may cause the presence of the C element. The N1s peak was found to be at 400.6 eV, while Ti 2p doublets were located at 464.4 and 458.4 eV corresponding to Ti 2p_3/2_ and Ti 2p_1/2_, which were approximately in accordance with the actual values. Subsequently, the O1s peak could be fitted into two peaks located at binding energies of 529.8 and 532.05 eV.

### 2.3. Morphology Analysis

**Figure 3:**
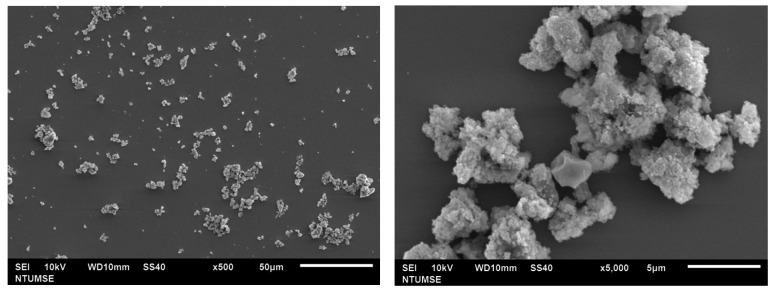
SEM micrograph of N-TiO_2_.

The morphology of doped TiO_2_ was observed using SEM and is shown in Figure 3. According to the results, N-TiO_2_ calcinated at 350 °C showed agglomerated clusters with spherical morphology. At high temperatures, particles tend to aggregate due to the particle growth process.

### 2.4. UV-Vis Absorption Spectra

**Figure 4:**
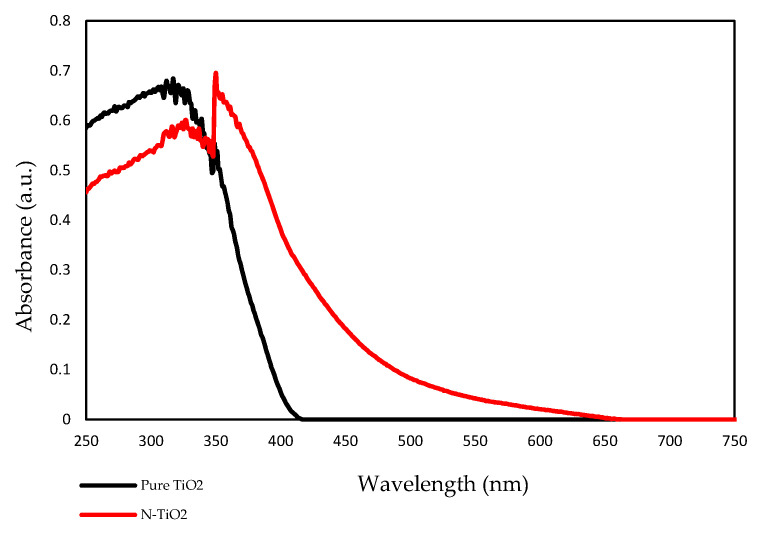
UV-Vis absorption spectrum of pure TiO_2_ and N-TiO_2_.

The optical property of the prepared TiO_2_ photocatalyst was measured using UV-vis spectroscopy. Figure 4 shows the UV-Vis absorption spectra of pure TiO_2_ (undoped) and N-TiO_2_. According to the results, pure TiO_2_ had an absorption edge around 390 nm, while the absorption edge of N-TiO_2_ was found to be around 450 nm. This shift toward the visible light in the absorption spectra of N-TiO_2_ was due to the incorporation of nitrogen into the TiO_2_ lattice, eventually leading to band gap narrowing.

### 2.5. Color Analysis of Tooth Bleaching

**Figure 5:**
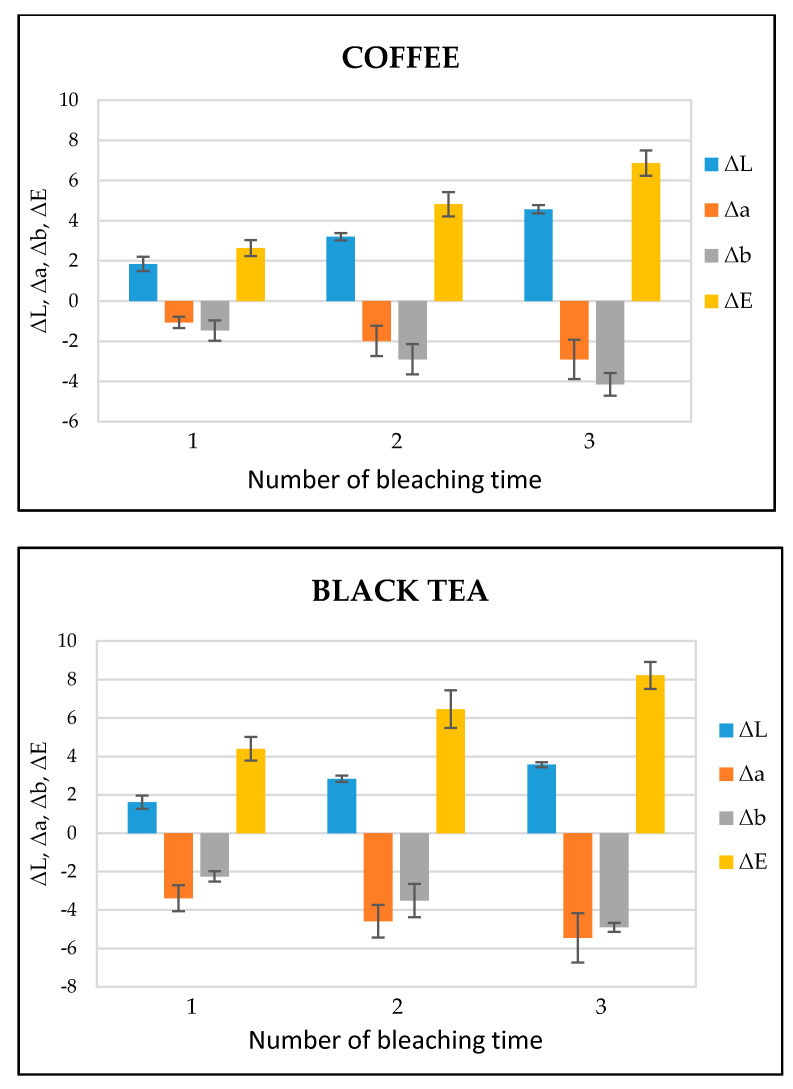
ΔL, Δa, Δb, and ΔE values of coffee- and black tea-stained bovine tooth samples treated with dental bleaching gel (*n* = 6).

Figure 5 shows the ΔL, Δa, Δb, and ΔE values of each bleaching time in the coffee- and black tea-stained bovine teeth groups, respectively. ΔL, Δa, and Δb stand for the difference of L, a, and b between baseline and each bleaching time while the color difference (ΔE) was calculated according to the formula ΔE = [(ΔL)^2^ + (Δa)^2^ + (Δb)^2^]^1/2^. After the bleaching treatment, the values of ΔL and ΔE gradually increased in both the groups, while the values of Δa and Δb exhibited a decrease. According to the statistical analysis, ΔE in both the groups showed significant differences (*p* < 0.05) between bleaching times (Table 1). The photographs of a representative image of tooth bleaching from black tea and coffee stained groups at the baseline and 1, 2, 3 times of bleaching is shown in Figures S1 and S2 respectively.

### 2.6. Biological Assays

**Figure 6:**
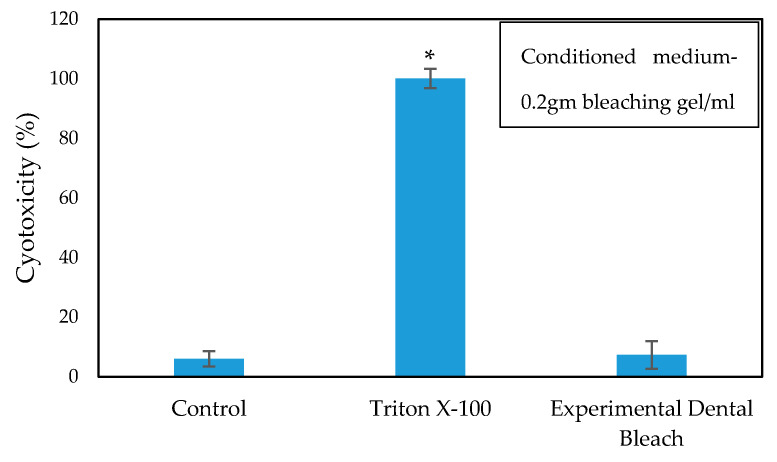
Cytotoxicity of the cells cultured in dental bleach-conditioned medium (*n* = 6), * *p* < 0.05 compared to the control group by student *t*-test.

**Figure 7:**
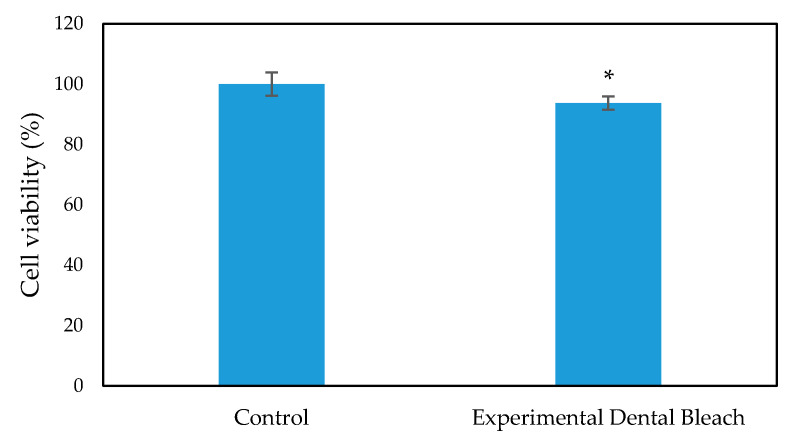
WST-1 assay for 3T3 cell viability evaluation on exposure to dental bleach-conditioned medium (*n* = 6), * *p* < 0.05 compared to the control group by student *t*-test.

The cytotoxicity and biocompatibility of the bleaching gel were determined via LDH and WST-1 assay, respectively, and the results are presented in Figure 6 and Figure 7, respectively. WST-1 is a colorimetric cell proliferation assay used to measure the biocompatibility while the LDH assay determines the cellular cytotoxicity. 3T3 cells were used in this study. Conditioned medium containing 0.2 g of bleaching gel (10% CaO_2_ + 1% N-TiO_2_ + 1.5% methylcellulose) per mL was prepared and used in this study. The WST-1 result showed a cell viability of 93.7% at 24 h while the LDH assay showed very low cytotoxicity of 7.3% when exposed to dental bleach-conditioned medium for 24 h. No significant toxicity was observed in 3T3 cells when cultured with the experimental bleaching gel.

## 3. Discussion

The study focused on the bleaching efficiency and cytotoxicity of the formulated dental bleaching gel using a discolored bovine tooth model. In view of the existing literature, there have been several in vitro models appointed for evaluating the efficacy of the dental bleaching agent, the important ones being the human or bovine tooth model, either cut or whole [26,27,28]. In this current study, bovine teeth were used as an in vitro model because of its similarity with human teeth in terms of physical and chemical properties, such as hardness, permeability, and density of the dentin tubule. Moreover, the collection of extracted human teeth is difficult for experiments with large sample sizes, and the consistency in the results may be affected by various factors, such as shade, age, thickness of enamel, and mineralization of the extracted human tooth surface [29].

In this study, discoloration of the bovine teeth was achieved by tea and coffee staining solutions, respectively, due to their cost effectiveness, easy preparation, and regular use by the majority of the population [30,31]. Shade change of the teeth after bleaching can be evaluated using several methods, such as visual comparison of the tooth surface with the standard tooth shade card or parametric analysis using a chroma meter. In this study, a chroma meter was used to evaluate the tooth shade as it can provide quantitative analysis in color matching, so that statistical analysis can be easily achieved and is more accurate than the visual analysis using a tooth shade guide.

The main and effective attraction of in-office dental bleaching products is the use of H_2_O_2_ as an active ingredient, but due to the diverse side effects of using high-concentration H_2_O_2_ as mentioned before, many researchers have now directed their research towards finding alternatives to control high-concentration H_2_O_2_ to provide effective bleaching without harm to the dental tissues. Therefore, CaO_2_, used as a bleaching agent in this study, is expected to be a potential alternative as compared to the other commercial agents with high concentrations of H_2_O_2_ (20–40%). H_2_O_2_ released from CaO_2_ can be controlled with the alterations in temperature and pH. Therefore, the release rate of H_2_O_2_ was controlled by adjusting the pH of the bleaching gel to 7. Moreover, the neutral pH of the dental bleaching products has been reported to be safe on enamel because it does not alter the surface roughness of the tooth even after several applications [10]. CaO_2_ is also capable of reacting for a longer duration in the medium, making its functionality even more efficient.

In previous studies, it was demonstrated that the efficiency of H_2_O_2_-based bleaching products was increased with the addition of visible light-activating TiO_2_ when exposed to visible light [32,33]. Therefore, in this study, N-TiO_2_ was used as a photocatalyst to increase the efficiency of CaO_2_-based bleaching gel. The nitrogen doping in the TiO_2_ lattice results in the formation of a new electronic state above the valance band, which leads to the shift in the absorption spectrum of N-TiO_2_ from UV light to the visible light region as analyzed by the UV-vis spectrophotometer (Figure 4). The formation of a new electronic band led to a decrease in the band gap energy value and was calculated by the Tauc plot method. The band energy value for pure TiO_2_ was found to be 3.2 eV while on the other hand, the nitrogen-incorporated TiO_2_ lattice exhibited a decrease in the band gap value from 3.23 eV to 2.85 eV. Moreover, to investigate the effect of nitrogen doping on the crystal phase of TiO_2_, XRD was performed. The phase composition of N-TiO_2_ contains major intensity of the anatase phase and minor rutile phase, similar to that of pure TiO_2_ (Figure 1). In view of the existing literature, the anatase phase is a better photocatalyst than the rutile phase [34]. Therefore, in the present study, it was important to retain the intensity of the anatase phase in N-TiO_2_ in order to fabricate a strong photocatalyst to make a stronger dental bleach. Additionally, the results of XPS analysis (Figure 2b) confirmed the existence of N in the TiO_2_ lattice along with the elements Ti, O, and C. The observed N1s peak with binding energy at 400.1 eV is assigned to N-O-Ti linkage, i.e., nitrogen bonded to oxygen sites (interstitial doping). The high nitrogen content results in an effective visible light-activating photocatalyst even though there is slight particle aggregation at high temperatures due to the particle growth process.

The bleaching efficiency of the experimental bleaching gel was determined by comparing the color values at each bleaching time. The important indicator for the bleaching evaluation is the determination of the L value; the higher the L value, the brighter or whiter the teeth appear. In the present study, the value of L increased with each bleaching time in both the groups, indicating the brightness or whiteness was upgraded with each consecutive bleaching. On the other hand, the total color difference, i.e., ΔE, gradually increased during each bleaching treatment. ΔE is categorized into six ranks: 0.5 or less ΔE value, wherein macroscopically no color difference was seen; between 0.5 and 1.5, a small difference was observed with effort to the naked eye; between 1.5 and 3.0, a slight difference was clearly noticed; 3.0–6.0 values, when a substantial difference was seen; between 6.0 and 12.0, a marked difference was observed; and 12.0 or higher, when a different color line was noticed [35]. In this study, ΔE after the last post-treatment was in the range of 6.0–12.0, representing a significant difference (Figure 5).

Successful bleaching treatment brightens the teeth effectively without compromising safety. Therefore, a cytotoxicity test was conducted in order to evaluate the safety and biologic properties of the bleaching gel. We found that the bleaching components were non-toxic to 3T3 cells (Figure 6 and Figure 7). No significant difference was observed between the experimental group and control group. Therefore, the biocompatibility of the experimental bleaching gel was good when tested on 3T3 cell lines and should be safe in clinical use. Future study will focus on the quantification of released H_2_O_2_ from CaO_2_ and a more delicate mechanism under different conditions.

## 4. Materials and Methods

### 4.1. Preparation of N-TiO_2_

An organic nitrogen source urea was used for nitrogen doping. Briefly, 3M urea solution was prepared, and titanium (IV) oxide (21-nm primary particle size, Sigma-Aldrich, St. Louis, MI, USA) was dispersed in it at a ratio of 0.1 g/mL. The mixture was stirred for 24 h and later dried in the oven at 80 °C, followed by calcination in a furnace at 350 °C for 2 h.

### 4.2. Characterization

The phase identification of pure TiO_2_ and N-TiO_2_ was carried out using an X-ray diffractometer (XRD) (TTRAX 3, Rigaku, Tokyo, Japan). The surface composition and electron binding energy of TiO_2_ and N-TiO_2_ was measured using an X-ray photoelectron spectroscopy (XPS) (Theta probe, Thermo Scientific, Waltham, MA, USA). The morphology was observed using a scanning electron microscope (SEM) (JSM6510, JEOL, Tokyo, Japan). The UV-Vis spectroscopy (CARY 300nc, Agilent, Santa Clara, CA, USA) was used to record the absorption spectra of the samples.

### 4.3. Preparation of Stained Teeth

Twelve freshly extracted bovine incisors purchased from a local meat market in Taipei, Taiwan were used in this study. After extraction, the teeth were dipped in hot boiling water for 20 s, and the soft tissues were removed using a scalpel. The enamel surface was polished with ascending grit silicon carbide papers starting from #100 up to #1000 under running water, removing 300 µm from the enamel surface to create a smooth and flat surface. The teeth were then stored in water at 4 °C.

Black tea and coffee stain solutions were prepared. Briefly, two black tea bags (Earl Grey Twinings) each weighing 2 g were immersed in 100 mL of boiling water for 5 min to prepare a black tea stain solution while the coffee stain solution was prepared by adding 4 g of ground coffee powder (Nescafe) in 100 mL of boiling water. The teeth were then divided into two groups (*n* = 6) and immersed into the respective above-mentioned staining solutions for a week in the incubator at 37 °C. The solutions were renewed after every 3 days and stirred once a day to avoid sedimentation.

### 4.4. Color Analysis

After the teeth were immersed in the staining solutions for a week, they were rinsed under tap water to remove excess coffee and tea from the surface, respectively, followed by drying using kimwipes. Prior to bleaching, the CIELAB values of the stained enamel surface were recorded as a baseline value using a dental chroma meter (VITA EasyShade Compact, Vident, Model # DEASYCBU, Yorba Linda, CA, USA).

### 4.5. Tooth Bleaching

The experimental bleaching gel was composed of 10% calcium peroxide, 1% N-TiO_2,_, and 1.5% methylcellulose as a thickener. The pH of the gel was adjusted to 7.0 before the application onto the test tooth surface.

A thin layer of the experimental bleaching gel was applied on the test tooth surface with a brush and irradiated for 1 min with a light emitting diode (LED) light curing device (LITEX 696, Dentamerica Asia Inc., Taipei, Taiwan). Following which, the bleaching agent was left for 5 min on the tooth surface, and the bleaching gel was then rinsed with fresh water and dried. A VITA EasyShade Compact chroma meter was used to measure the L, a, and b values of the bleached teeth after rinsing and drying of teeth, where L* represents the lightness of the sample from black (0) to white (100), a* represents the green-red coordinate, and b* represents the blue-yellow coordinate. The bleaching and color measurements were repeated thrice per tooth. The difference between baseline and each bleaching time for L, a, and b values were denoted as ΔL, Δa, and Δb, respectively. The color difference (ΔE) was calculated according to the equation below:

ΔE = [(ΔL)^2^ + (Δa)^2^ + (Δb)^2^]^1/2^

### 4.6. Biological Assays

#### 4.6.1. Cell Culture

The 3T3 cell line (mouse embryonic fibroblasts) (Bioresource Collection and Research Center, Taiwan) was used as a cell source in this study. Dulbecco’s modified Eagle’s medium (DMEM) (Sigma, USA) supplemented with 10% fetal calf serum (FBS) (Gibco, Gaithersburg, MD, USA) and 1% antibody (Gibco, USA) was used for the cell culture. The cells were incubated at 37 °C in a 5% CO_2_-containing atmosphere.

#### 4.6.2. Cell Viability

Cells were seeded in 96-well plates at a cell density of 1 × 10^4^ cells/well and incubated for 24 h at 37 °C. After 24h, the used DMEM was aspirated, washed with PBS, and then cultured with the conditioned medium for 24 h at 37 °C. The conditioned medium was obtained according to the ISO standard 10993-12:2012. Briefly, 5 mL of the conditioned medium was prepared (0.2 g bleaching gel per 1 mL of DMEM culture medium incubated for 30 min at room temperature). The conditioned medium was filtered through a sterile filter and subsequently used for cell culture.

Cell viability was evaluated using the WST-1 assay kit (Takara, Japan) according to the manufacturer’s instruction. The conditioned medium was replaced with WST-1 working solution for 2 h, and the absorbance of formazan, a colored dye produced by viable cells using WST-1, was measured at 450 nm by a microplate reader (Spectramax plus 384 microplate reader, Molecular Devices, CA, USA). Untreated cells, cultured with DMEM medium, were used as controls.

#### 4.6.3. Cell Cytotoxicity

The cytotoxicity of the prepared dental bleach was quantified using the LDH assay kit (Takara, Japan) according to the manufacturer’s instructions. The presence of LDH in the medium is an indicator of cellular toxicity. The conditioned medium after incubation with cells for 24 h was transferred and mixed with LDH working solution in a 1:1 ratio. The absorbance of LDH was measured at 450 nm, after incubating for 30 min in the dark using a microplate reader (Spectramax plus 384 microplate reader, Molecular Devices, CA, USA). The cells cultured with DMEM medium were used as controls, while the cells treated with medium containing 0.1% Triton X-100 were used as the positive control.

### 4.7. Statistical Analysis

After the calculation of ΔE data, it was subjected to statistical analysis and analysis of variance test (ANOVA) was performed, following which post hoc tukey’s test was used for comparison between the groups. A probability (*p*) value of 0.05 was considered statistically significant. The statistical difference between the control and experimental group in the WST-1 and LDH assay was evaluated by student’s t-test using GraphPad (Prism for Mac, GraphPaD software, San Diego, CA, USA).

## 5. Conclusions

In this work, nitrogen-doped titanium dioxide was successfully prepared and characterized. The synthesized N-TiO_2_ calcinated at 350 °C contained majorly the photocatalytic anatase phase and the absorbed nitrogen in the TiO_2_ lattice caused the band gap narrowing, resulting in visible light-activating N-TiO_2_. The dental bleaching gel comprising 10% CaO_2_ as an active ingredient and N-TiO_2_ as a photocatalyst demonstrated an efficient bleaching effect with a gradual increase in ΔL and ΔE on a coffee- and black tea-stained bovine tooth model in vitro, while the cell viability and cytotoxicity data provided an affirmation of the safety of bleaching gel. The overall findings of this study suggest the CaO_2_-based bleaching gel with N-TiO_2_ not only results in effective bleaching but can also decrease the potential side effects that are usually caused by a high-concentrated hydrogen peroxide-based dental bleaching procedure.

## Figures and Tables

**Table 1:** Mean ΔE and standard deviation.

Mean ΔE (SD)			
Solution	Times		
	1	2	3
Coffee	2.638 (0.392) ^a^	4.822 (0.601) ^b^	6.874 (0.629) ^c^
Black Tea	4.362 (0.589) ^a^	6.460 (0.973) ^b^	8.213 (0.705) ^c^

Different superscript lowercase letters in coffee and black tea rows indicate statistically significant differences among evaluation times in the same group.

## Data Availability

Not applicable.

## Supplementary Materials

The following are available online at https://www.mdpi.com/article/10.3390/ijms22073759/s1, Figure S1. Representative image of tooth bleaching in black tea stained group; Figure S2. Representative image of tooth bleaching in coffee stained group.
