# Supplementary material for: Nitrogen-Doped Titanium Dioxide Mixed with Calcium Peroxide and Methylcellulose for Dental Bleaching under Visible Light Activation
Source: Int J Mol Sci. 2021 Apr 4;22(7):3759. doi: 10.3390/ijms22073759 (PMC8038621; doi:10.3390/ijms22073759)
Supplement: Supplementary file 1 [file ijms-22-03759-s001.pdf]

<u>Supplementary File</u>

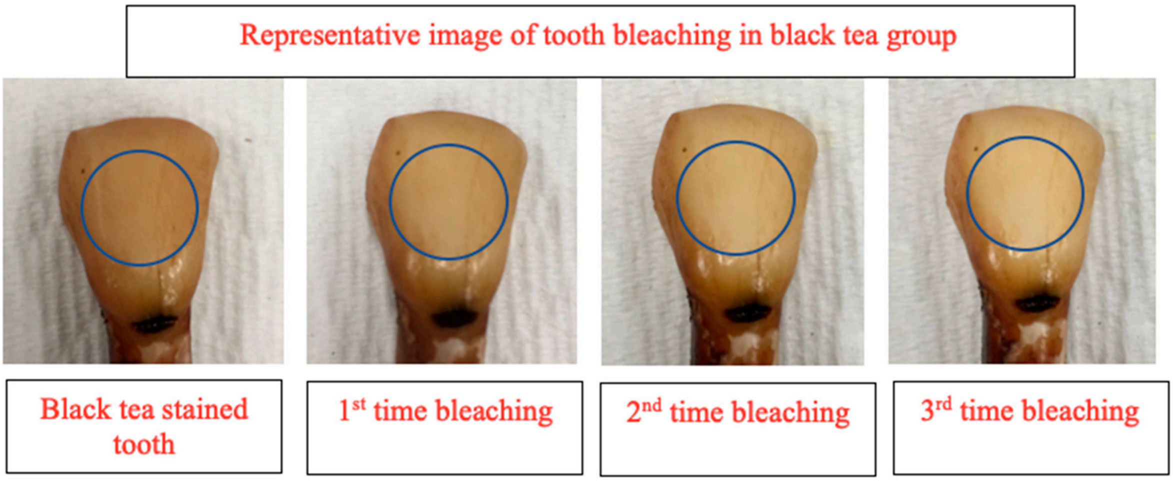

Supplementary figure 1. Representative image of tooth bleaching in black tea stained group

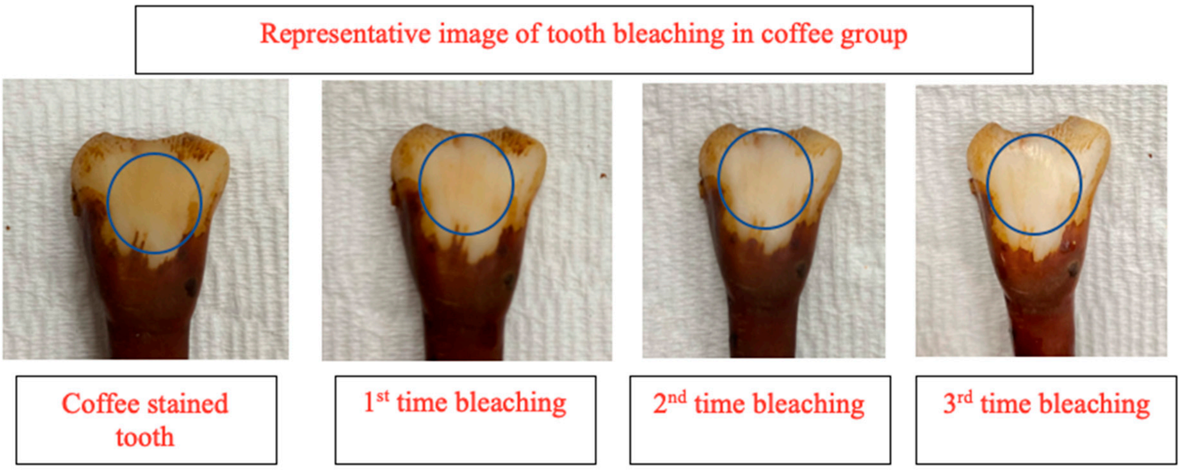

Supplementary figure 2. Representative image of tooth bleaching in coffee stained group
